# Aetiology of Diarrhoea and Virulence Properties of Diarrhoeagenic *Escherichia coli* among Patients and Healthy Subjects in Southeast Nigeria

**DOI:** 10.3329/jhpn.v28i3.5551

**Published:** 2010-06

**Authors:** E.I. Nweze

**Affiliations:** Department of Microbiology, University of Nigeria, Nsukka, Nigeria

**Keywords:** Diarrhoea, *Escherichia coli*, Virulence, Nigeria

## Abstract

Diarrhoeal diseases are one of the most important causes of illness and death all over the world. In Nigeria, the aetiology of diarrhoeagenic bacteria and the virulence of various *Escherichia coli* pathotypes have not been well-studied because most currently-published data were from the southwestern axis of the country. In total, 520 stool samples were collected from infants, young children, and other age-groups with acute diarrhoeal diseases in Enugu and Onitsha, southeastern Nigeria. Stool samples were collected from 250 apparently-healthy individuals, with similar age distribution and locality, who were considered control subjects. The stool samples were screened for diarrhea-causing bacterial agents. *E. coli* strains were isolated from both the groups and were examined by polymerase chain reaction (PCR) for 16 virulence genes. Of the 520 stool samples in the diarrhoea group, 119 (44.74%) were *E. coli*. Fifty (49.02%) were enteropathogenic *E. coli* (EPEC), 22 (21.57%) were enterotoxigenic *E. coli* (ETEC) while 7.84% was enteroaggregative *E. coli* (EAEC). Sex had no effect on the distribution of diarrhoeagenic bacteria, except for EIEC. The *E. coli* strains isolated from the diarrhoea and healthy asymptomatic age-matched control groups examined by PCR for 16 virulence genes indicate that the detection of EAEC, ETEC, EPEC, and EIEC was significantly associated with diarrhoea (p=0.0002). The study confirmed that several bacterial pathogens, such as *E. coli,* play an important role in the aetiology of acute diarrhoea in southeastern Nigeria. A routine surveillance, especially for diarrhoeagenic *E. coli*, would be useful in identifying outbreaks and help identify the potential reservoirs and transmission routes.

## INTRODUCTION

Diarrhoeal diseases and other related gastrointestinal disorders are one of the most important causes of illness and death all over the world, particularly among infants and young children (1–3). The major causes of diarrhoeal illness include, among others, limited access to or poor quality of water, poor food hygiene, and sanitation. The bacterial pathogens usually responsible for diarrhoeal illness include *Escherichia coli, Shigella, Salmonella, Campylobacter, Yersinia, Aeromonas,* etc. (4). Although in developed countries and in a few developing countries, the mortality rates have declined considerably in recent times due to improvement in general hygiene and advances in healthcare, the problem still persists in so many other countries where outbreaks of diarrhoeal diseases continue to affect millions of infants and young children (4, 5). The movement of persons within the same region and from one country to another increases the chance of transmission, thus requiring that comprehensive and first-hand information on peculiar situations in each locality or region be known.

Five major classes of diarrhoeagenic *Escherichia coli* (DEC) are associated with diarrhoeal diseases. These are: enterotoxigenic *E. coli* (ETEC), enteroinvasive *E. coli* (EIEC), enteropathogenic *E. coli* (EPEC), enterohaemorrhagic *E. coli* (EHEC), and enteroaggregative *E. coli* (EAEC). Some authors have proposed a sixth group: diffuse-adhering *E. coli* (DAEC) but this has not been clearly established (4, 6). Each of the several classes of DEC is defined based on the distinct virulence characteristics. Similarly, tests for these characteristics have been developed to distinguish DEC classes from each other and from non-pathogenic *E. coli* strains of the normal flora (7, 8). The epidemiological significance of each *E. coli* category in childhood diarrhoea varies with the geographical area. As expected, it has become clear that there are important regional differences in the prevalence of different categories of DEC. Several studies on the incidence of diarrhoeal illnesses caused by different classes of DEC have been conducted mainly in Latin America, Africa, South and Southeast Asia, and the Middle East (1). Study of the prevalence of DEC categories and their importance in diarrhoea has not been carried out extensively in Nigeria because only the southwestern axis has been well-studied, leaving the northern and southeastern areas (9–11).

Nigeria is a highly-populated country comprising so many cities having people of diverse socioeconomic and religious background. Enugu and Onitsha in the southeast flank are well-populated towns due mainly to the level of commercial activities going on in these cities. For instance, several people come to the city of Onitsha from other countries in West and East Africa and even beyond for one kind of commercial activity or the other. Sporadic endemic diarrhoea among adults and young children, however, contributes to the overall loss of productivity in developing countries and increases the risk that pathogens will be passed on to susceptible children or visitors. Therefore, to ascertain the level/spectrum of bacterial pathogens and to define the association of various categories of *E. coli* with diarrhoea in Enugu and Onitsha, Southeast Nigeria, a controlled study using the traditional culture/serology technique and polymerase chain reaction (PCR) for the identification of specific virulence factors was undertaken.

## MATERIALS AND METHODS

### Collection of samples

In total, 520 stool samples were collected from infants and other age-groups (age 0–45 years), with cases of acute gastroenteritis attending both private and public hospitals/medical laboratories in Enugu and Onitsha in Southeast Nigeria. These patients presented varying kinds of symptoms, such as nausea, vomiting, fever, loss of appetite, abdominal pains, malaise, and watery stool. In other words, patients were enrolled in the study if they had diarrhoea characterized by frequent watery stools (>3 times per day) with or without blood or mucus and had not taken any antimicrobial agent in the week preceding sampling. Patients with concomitant infections were excluded from the study. Most subjects were from low-income families and had no access to appropriately-treated potable water. A control group of 250 apparently-healthy subjects with a similar age distribution was selected from the same population. These subjects had non-diarrhoeal illnesses and had no history of diarrhoea and/or of taking any antimicrobial agent for at least one month.

### Isolation and identification

All the stool samples were inoculated on deoxycholate citrate, MacConkey, *Salmonella, Shigella* and thiosulphate-citrate bile salt-sucrose agars. They were also inoculated on selenite-F broth. After aerobic incubation at 37°C for 18–24 hours, the plates were observed for bacterial growth. Suspicious colonies were further subcultured onto Kligler-iron agar and motility-indole-urea medium.

Further identification of isolates was done using standard biochemical methods as described by Cowan and Steel (12). Serological identification of ETEC, EIEC, EPEC, and O157H7 was first done using Wellcome diagnostic antisera as put forward by Taylor (13). Heat-labile enterotoxin (LT) test and heat-stable enterotoxin (ST) test were performed as described by Evans *et al.* (14) and Giannella (15) respectively. Invasive test was done as described by Sereny (16). The DEC strains were later confirmed by PCR but only in patients whose stools grew *E. coli*. To detect/confirm EHEC, ETEC, EIEC, and EPEC, PCR assays were performed using the protocol described by Presterl *et al.* (4). Sixteen virulent genes were searched for: EPEC *eae, tir*, and *bfpA*; ETEC e*lt*, e*sth, and ingA*; EIEC *inv* and *sen*; EHEC *stx*1, *stx*2, *eae,* and *hly*; and EAEC *aggA, PAA, aaFA, set,* and *sen*. EAEC was detected using the cell culture technique, and five PCR assays were carried out. DNA was extracted from whole organisms by boiling. Bacteria were harvested from an overnight broth culture, suspended in 1 mL of sterile water and incubated at 100 °C for 10 minutes. The lysate was centrifugated, and 1 μL of the supernatant was used in the PCRs. For the PCR mixes, each of the primers was used at a concentration of 0.1 mM, with 0.2 mM of each deoxynucleoside triphosphate, 10 mM of Tris-HCl, 50 of mM KCl, 1.5 mM of MgCl_2_, and one unit of *Taq* DNA polymerase (Roche, Germany). The HEp-2 adherence assay was used for detecting EAEC. The patterns of adherence of EAEC were examined by the method described by Nataro *et al.* (4). Briefly, HEp-2 cells were grown overnight in 50% confluent monolayers on glass cover slips in 24-well tissue culture dishes. After the spent medium was discarded, 50 μL of overnight Luria broth bacterial culture plus 1 mL of fresh Eagle's minimal essential medium (GIBCO/BRL, Gaithersburg, MD) with 0.5% D-mannose was added to each well and incubated for three hours at 37 °C in an atmosphere of 5% CO_2_. Cells were washed twice after incubation with phosphate-buffered saline, fixed with 70% methanol for five minutes and stained with 10% Giemsa for 15 minutes. Cover slips were examined under an oil immersion light microscope for the characteristic stacked brick aggregation on HEp-2 cells by two microscopists. The control isolates included the following reference strains: RKI 17-2 for EAEC, DSM 8698 for EPEC, and ATCC 25922 for non-pathogenic *E. coli*.

### Statistical analysis

The effects of sex and age on the incidence of diarrhoeal diseases were tested for significance at 5% level using the chi-square test of homogeneity. The recovery of pathogens from the subjects with diarrhoea and the controls was compared by a two-tailed chi-square test (17).

## RESULTS

Of the 520 stool samples investigated, 228 (25.10%) yielded bacterial isolates that cause diarrhoeal diseases. Table 1 shows the isolation rates of the bacterial pathogens distributed according to sex groups. *E. coli* had the highest incidence rate (44.74%) while *Campylobacter* species had the lowest incidence rate (1.75%). The incidence of different *E. coli* strains encountered in the study according to sex groups showed that EPEC accounted for 30 (29.41%) of the total *E. coli* isolates, with 14 (46.67%) males and 16 (53.33%) females. Twenty-two (21.57%) ETEC strains were isolated from 12 (54.55%) males and 10 (45.45%) females. Fifteen (14.71%) strains of EIEC were also isolated from four (26.67%) males and 11 (73.33%) females. Twenty-eight (27.45%) EAEC isolates were recovered—10 (35.71%) from males and 18 (64.29%) from females. For EHEC, only seven (6.86%) isolates were recovered—three (42.86%) from males and four (57.14%) from females. Overall, 45 (44.12%) of the 102 isolates were males while 57 (55.88%) were females. The breakdown of the 30 EPEC strains according to serotype and age showed that six isolates of serotype O55 (3 from males and 3 from females) were isolated. There were five O26 strains (2 from males and 3 from females); O111 had four (3 from males and 1 from a female); O119 had two with one each from either sex; O125 had three (2 from males and 1 from female); O126 had two isolates, all from males. Serotype O127 and O129 had two isolates each with one each from either sex while O128 had three isolates, with one and two isolates from male and female population respectively. Only one of serotype O142 was isolated from a male.

**Table 1.** Sex distribution of diarrhoegenic bacteria isolated from stools of patients with gastroenteritis in Enugu and Onitsha

Pathogenic bacterial isolate	Isolates		No. of isolates from males	No. of isolates from females
	No.	%		
*Escherichia coli*	102	44.74	47	55
*Salmonella* spp.	36	15.79	12	24
*Shigella* spp.	35	15.35	23	12
*Klebsiella* spp.	21	9.21	11	10
*Aeromonas* spp.	15	6.58	6	9
*Plesiomonas* spp.	9	3.95	5	4
*Yersinia* spp.	6	2.63	4	2
*Camphylobacter* spp.	4	1.75	1	3
Total	228 (100)		109 (47.81)	119 (52.19)

In summary, there were 17 (56.67%) isolates of EPEC from males and 13 (33%) from females. For the ETEC strains, serotype O6 and O20 had two isolates, with one isolate recovered from either sex. Serotype O15 and O148 had three, each, with two from males and one from a female. Serotype O25 had four isolates (1 from a male and 3 from female); O128 had five isolates (4 from male and 1 from a female); O153 had two with all from the female while O159 had the only isolate from the male.

The summary shows that 12 (54.55%) of the 22 isolates were from males while 10 (45.45%) were from females. For the EIEC, there were 15 isolates comprising four serogroups: O28, O29, O124, and O136. Four (26.67%) were recovered from males while 11 (73.33%) were from females. Likewise, EHEC had seven isolates comprising four serogroups: O26, O111, O138, and O157. Only three (42.86%) were recovered from males while four (57.14%) were from females. Table 2 shows the enteric bacteria isolated from stool samples of the diarrhoea and control groups while Table 3 shows the age distribution of bacterial pathogens isolated from the diarrhoeagenic patients.

**Table 2.** Enteric bacteria isolated from stool samples in Enugu and Onitsha subjects

Bacterial isolate	Diarrhoea cases (n=520)		Controls (n=250)	
	No.	%	No.	%
EPEC	30	5.8	1	0.4
EAEC	28	5.4	2	0.8
ETEC	22	4.2	1	0.4
EIEC	15	2.9	2	0.8
EHEC	7	1.3	0	0
*Salmonella*	36	6.9	1	0.4
*Shigella*	35	6.7	0	0
*Klebsiella*	21	4.0	2	0.8
*Aeromonas*	15	2.9	2	0.8
*Plesiomonas*	9	1.7	1	0.4
*Yersinia*	6	1.2	2	0.8
*Campylobacter*	4	0.8	0	0
Total	228	43.8	14	5.6

EAEC=Enteroaggregative *E. coli*;

EHEC=Enterohaemorrhagic *E. coli*;

EIEC=Enteroinvasive *E. coli*;

EPEC=Enteropathogenic *E. coli*;

ETEC=Enterotoxigenic *E. coli*

**Table 3.** Age distribution of bacterial pathogens insolated form diarrhoeagenic patients in Enugu and Onitsha

Age-group (Years)	No. of patients	No. of isolates	EPEC	LT/ST	ETEC LT only	ST only	EIEC	EAEC	EHEC	*Shigella* spp.	*Salmonella* spp.	*Klebsiella* spp.	*Aeromo nas* spp.	*Plesiomonas* spp.	*Yersinia* spp.	*Campylobacter*
0–4	200	89	25	0	0	2	0	22	0	4	5	3	2	0	0	0
5–14	121	61	5	2	1	1	1	6	2	15	17	7	4	1	1	1
15–30	101	38	0	2	2	2	9	0	3	8	10	5	6	2	1	2
31–45	57	24	0	2	1	1	2	0	1	5	3	4	2	3	2	1
>45	41	16	0	3	1	2	3	0	1	3	7	2	1	3	2	0
Total	520	228	30	9	5	8	15	28	7	35	36	21	15	9	6	4

EAEC=Enteroaggregative *E. coli*;

EHEC=Enterohaemorrhagic *E. coli*;

EIEC=Enteroinvasive *E. coli*;

EPEC=Enteropathogenic *E. coli*;

ETEC=Enterotoxigenic *E. coli*;

LT=Heat-labile enterotoxin;

ST=Heat-stable enterotoxin

In total, 102 of *E. coli* strains were evaluated by PCR to detect 16 virulence genes to classify the DEC categories. ETEC, EPEC, EIEC, and EHEC were defined as strains having the respective genes. EAEC strains were identified by their ability to adhere to HEp-2 monolayers in an aggregative pattern. There was considerable heterogeneity of the EAEC isolates with respect to virulence gene content. The incidence of each virulence gene among *E. coli* isolates in the diarrhoea and control groups is shown in Table 4. The incidence of each virulence gene was significantly higher in the diarrhoea group than in the control group. The incidence of DEC in the diarrhoea group (19.62%; 102 of 520) was significantly higher than that in the control group (2.4%; 6 of 250) (p<0.0001). The most prevalent category was EPEC, followed by EAEC, ETEC, EIEC, and EHEC. All the 30 EPEC strains were positive for the *eae* and *tir* genes; 13 of them were positive for the *bfpA* gene in the diarrhoea group. The 30 strains with *eae* and *bfpA* genes showed localized adherence to HEp-2 cells which is very typical of EPEC. Of the four virulent/targeted genes in the EHEC pathotype, only the Shiga toxin 1 (*stx*_1_) was not recovered. Among the ETEC strains, only the *lngA* was not isolated from the diarrhoeal subjects but from the control group against the LT and ST genes which had 14 and 18 isolates respectively (Table 4).

**Table 4.** Incidence of each virulence gene among *E. coli* isolates in diarrhoeal and control groups

Pathotype	Target/virulence gene	Virulence factor encoded by target gene	Diarrhoea (n=520)	Control (n=250)
EPEC	eae	Intimin (LEE-encoded adhesin)	17	0
	*bfpA*	Structural subunit of bundle-forming pilus	13	1
EHEC	*eae*	Intimin	3	0
	*hly*	Enterohaemolysin	2	0
	*stx*1	Shiga toxin 1	0	0
	*stx*2	Shiga toxin 2	2	0
ETEC	*elt*	LT	14	0
	*esth*	ST	8	0
	*lngA*	Longus pilus	0	1
EIEC	*inv*	Invasion plasmid	9	2
	*sen*	*Shigella* enterotoxin 2	6	0
EAEC	*PA*A	Aggregative adherence plasmid	10	1
	*aggA*	Aggregative adherence fimbriae 1	5	0
	*aafA*	Aggregative adherence fimbriae 2	7	1
	*se*t	*Shigella* enterotoxin 1	5	0
	*sen*	*Shigella* enterotoxin 2	1	0

EAEC=Enteroaggregative *E. coli*;

EHEC=Enterohaemorrhagic *E. coli*;

EIEC=Entero invasive *E. coli*;

EPEC=Enteropathogenic *E. coli*;

ETEC=Enterotoxigenic *E. coli*;

LT=Heat-labile enterotoxin;

ST=Heat-stable enterotoxin

## DISCUSSION

In Nigeria and many developing countries, diarrhoea caused by bacterial pathogens, especially *E. coli,* remains one of the major causes of morbidity and mortality among infants and young children (9, 11, 18). In developed and a few developing countries, recent improvements in biological techniques have drastically increased the rate of diagnosis/isolation of bacterial pathogens and consequently reduced the global death rate due to bacterial diarrhoea diseases (1). However, the situation in Nigeria still calls for more concern. This is because the incidence of diarrhoea is still high in Nigeria.

The present study, the first of its kind in southeastern flank of Enugu and Onitsha, Nigeria, implicated *E. coli* with the highest incidence of 44.74% with respect to diarrhoea. The majority of the previous studies in most countries of the world also implicated *E. coli* as the predominant bacterial agent in diarrhoeal diseases (19, 20). An exception to this report, however, was obtained by some researchers in Saudi Arabia, which reported *Shigella* as having the highest incidence among other bacterial pathogens causing diarrhoeal disease in that country (20).

Of the DEC groups, EPEC was recorded to have the highest incidence of 30 (5.8%) of the 102 strains isolated in the study, with serotype O55 predominating over other serotypes. Sex had no significant effect (p>0.05) on both distribution of various pathogenic bacterial isolates and various *E. coli* groups and serotypes encountered in the study. The reason for this is not known. However, the result is in agreement with the results of other researchers (8, 9) with respect to the incidence of EPEC and serotype O55. There are, however, some differences in the general distribution of EPEC compared to the previous reports from both Lagos (Southwest) and Port Harcourt (South South) in Nigeria (19, 21). This is not surprising since earlier reports also indicated that the incidence of EPEC varies from one locality to another (19, 21) just like the author observed of dermatological infections in Nigeria (22). In fact, this established variability from locality to locality of these aetiologic agents was part of the reasons for trying to establish the aetiology of diarrhoea in Enugu and Onitsha, situated in the southeastern zone of Nigeria. The distribution of EPEC strains was significantly different (p<0.05) with respect to age-group and was mostly confined to the age-group of 0–4 years. Nevertheless, our study tested more persons in this age range. The implication of the results so obtained is that infants and children are at greater risk of contracting diarrhoea-related problems due to EPEC. This observation was reported previously in Lagos (9).

The sex distribution of the overall bacterial isolates shows that *E. coli, Salmonella* spp., and *Aeromonas* spp. are more predominant among females while the remaining other isolates were higher in males. Some of these differences were quite significant (p<0.05). Results of previous studies in Ibadan, Ile-Ife, and Lagos (all in southeastern Nigeria) suggest that the incidence of ETEC is higher among males than among females (19).

The present study agrees with their observations, although the figure obtained in this study is not statistically significant (p>0.05). Similarly, this same situation holds for the remaining other *E. coli* strains, EPEC, and EHEC where their individual distributions among sex-groups were not statistically significant (p>0.05). Conversely, EIEC strains seem to be more predominant among females (p>0.05) than among males. EIEC, EHEC, and ETEC (except for heat-stable strains) were not isolated from children aged less than five years. The reason may be due to the protection obtained from their mothers, which is normally secreted through breastmilk.

In Nigeria, nursing mothers are always encouraged to undergo exclusive breastfeeding of their babies for at least six months after delivery, and this could be one of such benefits of this programme.

EAEC was found only in those aged 0–14 years. This observation contrasts the report of Okeke *et al.* (11) where only EHEC and EAEC were significantly associated with diarrhoea (p<0.05). EHEC appears to be the least *E. coli* strain isolated in our study, with the incidence of 6.68%. The reason for this observation is not known, although it is in agreement with an earlier observation in Lagos, southern Nigeria (9).

Virulence locus probes and HEp-2 adherence were used for examining in details the properties of all DEC organisms isolated during the study. This study has shown that all the DEC groups are important diarrhoeal pathogens among young children and adults in Southeast Nigeria. Of all DEC pathotypes, EPEC was the most isolated followed by EAEC, ETEC, EIEC, and EHEC in that order. This finding on EPEC is in contrast to that reported in southern Nigeria (11); however, the same report agrees with the data in this study with respect to EAEC. Although EHEC was the least encountered in our study, it is contrary to the report obtained from southern Nigeria. Outbreaks and sporadic cases of EHEC have been reported in developed countries of North America, Japan, Europe, and even Australia. However, there have been a few reports of sporadic EHEC in African countries. Three large outbreaks of EHEC were previously reported in Swaziland, Central African Republic, and the Cameroon (23–25) but some authors criticized the methodology used in those studies as being non-specific or insensitive (11). Despite this, the findings tend to align with the earlier observation that EHEC may be rare after all. To buttress this, no strain of any EHEC from the stool of control subjects compared to the other DEC groups was isolated. This may be a good news for those in southeastern part of Nigeria, since the presence of this pathogen presents a potential treatment problem as it is sometimes treated empirically with antibiotics which normally can lead to a poor prognosis by precipitating haemolytic-uraemic syndrome in EHEC-infected individuals (11, 26). Nevertheless, as other bacterial diarrhoeagenic pathogens were recovered, therapy with antibiotics is still an option, making the need for better diagnosis very important. EAEC was the most prevalent DEC after EPEC. EAEC has emerged as a significant agent of diarrhoea all over the world (27). In addition, EAEC was previously reported to be endemic in southern Nigeria and in sub-Saharan Africa (11, 28, 29); so, the observation on the occurrence of EAEC concurs with what was reported in southwestern Nigeria and elsewhere, especially in sub-Saharan Africa. Just like other researchers observed (11, 30) and which appears typical with EAEC, the virulent gene content belonging to this DEC group identified in this study was heterogeneous. EAEC causes growth defects in children and, thus, constitutes a burden of disease (11).

It will be useful if the risk factors and transmission patterns could be ascertained in the two cities under study so as to forestall future outbreak and occurrence of this DEC pathotype. Future studies should, thus, focus on revealing and identifying the risk factors and transmission routes for these emerging pathogens within the study area and even beyond.

Microbiological surveillance and diagnosis of infections caused by EPEC, EHEC, EIEC, ETEC, EAEC, and related bacterial organisms need significant improvement in the present diagnostic capabilities of local laboratories within the region. If such diagnostic resources are made available, even only in teaching hospitals (tertiary-care institutions), these could be instrumental in identifying outbreaks. Some authors suggested that educating the residents on the benefits of good personal hygiene, good sewage and refuse-disposal methods, and infection-control strategies can help reduce the scourge of diarrhoea (4, 20). This remains to be seen.

## ACKNOWLEDGEMENTS

The author is grateful to Mr. Dubem Eze, a medical laboratory scientist in Onitsha, for his valued assistance, especially in collection of samples.
